# Estimating adjusted prevalence ratio in clustered cross-sectional epidemiological data

**DOI:** 10.1186/1471-2288-8-80

**Published:** 2008-12-16

**Authors:** Carlos Antônio ST Santos, Rosemeire L Fiaccone, Nelson F Oliveira, Sérgio Cunha, Maurício L Barreto, Maria Beatriz B do Carmo, Ana-Lucia Moncayo, Laura C Rodrigues, Philip J Cooper, Leila D Amorim

**Affiliations:** 1 State University of Feira de Santana, Feira de Santana, Brazil; 2 Department of Statistics, Federal University of Bahia, Salvador, Brazil; 3 Instituto de Saúde Coletiva, Federal University of Bahia, Salvador, Brazil; 4 London School of Hygiene and Tropical Medicine, London, UK; 5 Instituto de Microbiologia, Universid San Francisco de Quito, Quito, Ecuador

## Abstract

**Background:**

Many epidemiologic studies report the odds ratio as a measure of association for cross-sectional studies with common outcomes. In such cases, the prevalence ratios may not be inferred from the estimated odds ratios. This paper overviews the most commonly used procedures to obtain adjusted prevalence ratios and extends the discussion to the analysis of clustered cross-sectional studies.

**Methods:**

Prevalence ratios(PR) were estimated using logistic models with random effects. Their 95% confidence intervals were obtained using delta method and clustered bootstrap. The performance of these approaches was evaluated through simulation studies. Using data from two studies with health-related outcomes in children, we discuss the interpretation of the measures of association and their implications.

**Results:**

The results from data analysis highlighted major differences between estimated OR and PR. Results from simulation studies indicate an improved performance of delta method compared to bootstrap when there are small number of clusters.

**Conclusion:**

We recommend the use of logistic model with random effects for analysis of clustered data. The choice of method to estimate confidence intervals for PR (delta or bootstrap method) should be based on study design.

## Background

While the odds ratio (OR) is one of the most frequently used measures of association between a risk factor and an outcome in epidemiology, the risk ratio(RR) and prevalence ratio (PR) are important indices to quantify the strength of association between a given disease and a suspected risk factor [1]. The main reason for the popularity of the OR is because the OR is the measure of association usually provided by logistic regression models. There is a large body of literature discussing the relationship between OR and RR(or PR) [2,3] and an ongoing debate on the appropriateness of odds ratios versus prevalence ratios as measures of effect in cross-sectional studies [4-9]. It is known that the OR overestimates the RR(or PR) [3,10,11] when the outcome of interest is common (larger than 10%, for instance). The major limitation of using OR under such circumstances is related to its misinterpretation as PR [11].

The use of odds ratios in cross-sectional studies, a common practice among epidemiologists, has been criticized because prevalence odds ratios are good estimates of prevalence ratios only under specific circumstances [12-14]. More recent studies examining the differences between OR and PR, according to variations in the prevalence of exposure and disease, have shown that differences between odds ratios and proportions ratios, relative risks or prevalence ratios increase with increasing disease frequency [15]. There are several statistical models that can provide adjusted estimates for PR, including the logistic model, Poisson regression and log-binomial regression [3,10,16-18]. However, there is no consensus about the best approach to obtain the adjusted PR and these methods may lead to different conclusions. The main appeal of estimating PR as a measure of association is that PR is more easily interpreted than the OR in cross-sectional studies with common outcomes. For instance, a PR of 2 means that the proportion of cases among exposed is 2 times higher than among unexposed subjects, while an OR of 2 does not necessarily have the same meaning. Previous reports have estimated the PR in the context of simple random samples, where the responses of distinct individuals can be considered independent to each other [10], but in many situations this assumption may not be satisfied. Clustered cross-sectional studies have become increasingly popular in epidemiology, especially when the use of simple random sample designs is not feasible. In such case, the analysis must take into account the degree of similarity between subjects within clusters [19]. In the present paper we have evaluated methods for estimating adjusted PR in clustered cross-sectional studies using random-effects models.

The evaluation of these methods has been motivated by data from the SCAALA (Social Changes, Asthma and Allergy in Latin America Programme) studies in Brazil [20] and Ecuador [21], both of which use clustered data. The study in Brazil was conducted in Salvador, located in the Northeast of the country, and evaluated associations between the prevalence of asthma and other allergic diseases in children and potential risk factors, including living conditions and exposure to infections [20]. The participants of this study were recruited from 24 small geographical areas selected to represent the population without sanitation in the city of Salvador in 1997. The clustered study design could lead to dependence on asthma occurrence in the children living in the same geographical area. The Ecuadorian study was conducted in the province of Esmeraldas, one of the poorest regions of the country, to investigate the impact of long term treatment with the broad-spectrum anthelmintic drug, ivermectin, used for the control of onchocerciasis, on the prevalence and intensity of soil-transmitted helminth infections in school-age and pre-school children [21]. The data from this study was used to compare the prevalence of *Trichuris *infection between children living in treated and non treated communities.

In this paper, we have evaluated modeling strategies for the estimation of adjusted PRs in cross-sectional studies with common outcomes through simulation studies in the settings of clustered data.

## Methods

Logistic regression is the most popular model used for the analysis of binary outcomes to estimate adjusted odds ratios. These can be expressed in terms of the estimated effect of the factor of interest on the outcome, or more simply as the exponential of the factor's coefficient (for instance, *OR *= exp(*β*_1_), where *β*_1 _denotes this effect). The estimation of the PR, however, requires a more complicated mathematical expression that relates the effects and the values of the factors of interest. For example, suppose it is of interest to evaluate the effect of an exposure (*X*_1_) on the occurrence of an outcome while controlling for *k *- 1 confounders (*X*_2_,..., *X*_*k*_). In such circumstances, the PR between exposed and unexposed subjects could be expressed as:

$$PR=\frac{1+\exp\{-\beta_{0}-\beta_{2}X_{2}-\ldots -\beta_{k}X_{k}\}}{1+\exp\{-\beta_{0}-\beta_{1}-\beta_{2}X_{2}-\ldots -\beta_{k}X_{k}\}}$$

Note that the PR depends on the values of the covariates in the model. Some alternative models discussed in the literature that allow a simpler approach for the estimation of the PR include the log-binomial, the Poisson with robust variance estimator and the Cox model with the same follow-up time for all subjects [3,10,16-18]. A major limitation of the Poisson and the log-binomial models, however, is that they allow prediction of probabilities out of the interval [0, 1]. The log-binomial model fails to converge when this happens. Moreover confidence intervals obtained using Poisson and Cox models are wider than those obtained from log-binomial model, requiring the use of a robust variance estimator. According to Moineddin, Matheson and Glazier (2007) [22], direct conversion of adjusted OR into PR is impractical because the notions of linearity, confounding and interaction are not equivalent between the different models. Thus, the logistic regression model would appear to be an alternative approach for the estimation of PRs and their confidence intervals.

### Standardization Procedures

Several standardization procedures for epidemiologic measures of effect based on regression models have been proposed. Wilcosky and Chambless [23] following Lane and Nelder [24] referred to three approaches for adjusting the prevalence ratio estimated from a logistic regression model: the *conditional *method where a standard value, usually the mean, is chosen for the covariates and the prevalence is computed for each comparison group; the *stratified *method, where for each comparison group the prevalence is estimated as a weighted average of the strata defined by combination of covariates values with weights chosen from a standard population; and the *marginal *method where, for each comparison group, the prevalence is computed for every combination of values of the covariates and averaged over all observations. The stratified and marginal methods will give the same results if the weights are chosen as the relative sizes of strata in the study population. In addition, all observations in every stratum have identical values of the covariates similar to the *direct *standardization procedure, which is a weighted average of predictions of all strata formed by the covariates where the weights are taken from a reference population. As an example, we have data on *n *individuals with a dichotomous exposure *X*_1 _(1 = exposed, 0 = non-exposed), and one continuous covariate, *X*_2_. Using the conditional method, the adjusted PR is given by $PR=\frac{1+\exp\{-\beta_{0}-\beta_{2}{\bar{X}}_{2})}{1+\exp\{-\beta_{0}-\beta_{1}-\beta_{2}{\bar{X}}_{2}\}}$ where ${\bar{X}}_{2}$ represents the mean of *X*_2_. For the marginal method the adjusted PR is given by $PR=\frac{\frac{1}{n}\sum_{i}\left(1/\{1+\exp(-(\beta_{0}+\beta_{1}+\beta_{2}X_{2i}))\}\right)}{\frac{1}{n}\sum \left(1/\{1+\exp(-(\beta_{0}+\beta_{2}X_{2i}))\}\right)}$ where the summation is over all *n *individuals. A similar expression is used in the stratified method, with *k*, the number of strata formed by categorizing *X*_2_, replacing *n *and with the use of weights *W*_*k *_chosen from a reference population

$$PR=\frac{\sum_{k}w_{k}(1/\{1+\exp(-(\beta_{0}+\beta_{1}+\beta_{2}X_{2i}))\})}{\sum_{k}w_{k}(1/\{1+\exp(-(\beta_{0}+\beta_{2}X_{2i}))\})}$$

An alternative approach for conditional standardization is by specifying a reference value for each covariate rather than using their mean values [10]. This approach is particularly useful when considering several levels of exposure for a covariate.

Flanders and Rhodes [25] provide formulae for the estimated variance of the adjusted prevalence for all methods. In the next subsection we discuss methods to obtain confidence intervals using random effects logistic model for the setting of clustered data.

### Estimating Prevalence Ratio using Logistic Model with Random Effects

#### Logistic Model with Random Effects

There are two large families of statistical models that account for the correlation in different ways, leading to estimated parameters that have different interpretations, which are denoted as marginal models and random effects models [26]. We will focus on a well-established approach for modeling clustered/correlated data that introduces random effects in the model of interest. This approach allows the relationship between the outcome and the covariates to vary from one subject to another. The random effects models take into account adjustment on non-observed individual characteristics reflecting a natural heterogeneity across subjects. By using this approach, the correlation between the observations from the same analysis unit arises from their sharing specific but unobserved properties of the respective subject. A random effects logistic regression model can be used to predict binary outcomes when observations are correlated or come from clustered data. This method makes possible to deal simultaneously with the problems of correlated observations and measurement error in the dependent variable. For illustration, let *Y*_*ij *_be a dichotomous outcome at cluster *j *for subject *i *and *X*_1*ij *_and *X*_2*ij *_two covariates. The random effects logistic regression model can be written as

*logit*[*P*(*Y*_*ij*_|*X*_1*ij*_, *X*_2*ij*_, *u*_*oj*_)] = *β*_0 _+ *β*_1_*X*_1*ij *_+ *β*_2_*X*_2*ij *_+ *u*_*oj*_

where *u*_*oj *_~ *N *(0, *σ*^2^) represents a cluster specific random effect, leading to a random intercept logistic regression model, which is the simplest example of a generalized linear mixed model (GLMM). This model describes the combined effect of all omitted subject-specific covariates that cause some subject to be more prone to disease (for example) than others. It is appealing to model unobserved heterogeneity in the same way as observed heterogeneity by simply adding the random intercept to the equation.

Using the estimates for the effects (*β*'s) of covariates on the outcome obtained with the random effects logistic model, we can choose a standardization procedure and estimate PR using the formulae presented previously relating the values of covariates and their effects to the prevalence ratio. Several investigators interpret the regression coefficients (or the odds ratios) obtained from the logistic model with random effects in the same way as in the usual logistic regression model, by conditioning on the random effects [27-29]. According to Hardin and Hilbe (2003), when explicitly modeling the source of heterogeneity in the logistic regression with random effects, the fixed regression parameters have an interpretation for individuals, which is subject specific [30].

#### Confidence Intervals for Prevalence Ratios

Methods for obtaining large sample confidence intervals for prevalence ratios include the delta method and the bootstrap. The delta method is a general technique for asymptotic distributions of functions of random variables, based on Taylor series approximation [31], and the bootstrap is based on resampling the data with replacement and using the bootstrap replications to estimate the functions of interest [32]. Both methods are used to estimate the standard error of PR from the random effects logistic regression model. For the delta method, adjusted confidence intervals are given by $\exp(\log(\hat{PR})\pm z_{\alpha /2}\hat{se}(\log(\hat{PR})))$, where $\log(\hat{PR})$ is the estimate of the adjusted log(*PR*), $\hat{se}(\log(\hat{PR}))$ the estimate of the standard error of log(*PR*) and *z*_*α*/2 _is the quantile of the standard normal distribution. In the bootstrap estimation 1000 bootstrap replications are used to produce the bootstrap distribution of PR. The confidence interval is based on normal theory, assuming that log(*PR*) is normally distributed, which is often approximately the case in sufficiently large samples, and uses the bootstrap estimate of sampling variance. The bootstrap confidence intervals are given by $\exp(\log(\hat{PR})\pm z_{\alpha /2}\hat{se*}(\log(\hat{PR}*)))$, where $\log(\hat{PR}*)$ is the bootstrap estimate of the adjusted log(*PR*) and $\hat{se*}(\log(\hat{PR}*))$ is the bootstrap estimate of the standard error of log(*PR*). An alternative approach, called bootstrap percentile interval [33], uses the empirical quantiles of the bootstrap estimates to form the interval. The limits of the interval are given by the 2.5 and 97.5 percentiles, for example, if we consider a 95% confidence interval. Previous simulation studies have pointed out an equivalence between the delta and bootstrap methods in the analysis of independent observations [10]. We used a cluster bootstrap procedure in which clusters are selected by simple random sampling with replacement and there is no subsequent permutation [34]. The behavior of both methods for the clustered data setting is compared here via simulation described in a following subsection. We are going to integrate these approaches for the estimation of PR using random effects logistic regression.

### Epidemiological Studies

A brief description of two epidemiological studies whose data are used to illustrate the methods discussed in the paper is presented next. Both studies have outcomes with prevalence greater than 10% and are related to relevant health problems in children in developing countries.

#### SCAALA-Salvador Study

An epidemiological study is being conducted in the city of Salvador, in the Northeast of Brazil, to study the association between life conditions, immunological profile and occurrence of allergic diseases. The research project is called Social Changes and Asthma and Allergy in Latin America Programme (SCAALA). Information about asthma was obtained through the use of a Portuguese version of the questionnaire used by the International Study of Allergy and Asthma in Childhood (ISAAC) [35]. The design of this study has been reported elsewhere [20]. Briefly, children were recruited from 24 areas scattered around the city making this a clustered study. In this study, information of 1445 children aged 4 to 11 years-old was collected.

Because causes of asthma are incompletely understood and there has been a recent interest in the relationship between psychosocial factors and asthma [36,37], the aim of the analyses presented here is to investigate the impact of maternal mental health status on the occurrence of asthma in their children. The data used was collected in 2005 and included information on maternal mental health status and other maternal characteristics, such as educational level, smoking status and history of asthma, as well as child's characteristics, such as age, gender, and occurrence of asthma. The definition of maternal mental health status has been reported elsewhere [38]. Briefly, a self-reported questionnaire (SRQ) of 20 items was used for psychiatric screening of common minor mental disorders (depression, anxiety and other psycho-somatic dysfunctions) [39]. A cut-off point for the definition of probable cases of common minor mental disorders was defined as 8 or more positive answers, a definition that although not representing psychiatric diagnosis does indicate significant psychiatric suffering. For the analysis presented here, we considered data from 758 children and evaluated the impact of maternal mental health status in the occurrence of childhood asthma, controlling for child's age and gender, and maternal educational level.

#### SCAALA-Ecuador Study

Another important health problem throughout developing countries is parasite infections [40]. The National Program for Elimination of Onchocerciasis in Ecuador distributes ivermectin in endemic areas with the aim of eventually eliminating the infection from Ecuador. Ivermectin is a broad-spectrum anthelmintic drug that is efficacious for the treatment of geohelminth infections, including *Ascaris lumbricoides*, *Trichuris trichiura *and *Strongyloides stercoralis *[41]. To evaluate the effect of ivermectin on the epidemiology of these infections, a study was conducted with 3705 children aged 6–16 from rural afro-Ecuadorian communities in the province of Esmeraldas, Ecuador. The children were selected from 31 communities that have been treated with ivermectin and from other 27 adjacent villages, which were matched with ivermectin-treated communities by ethnicity, social and economic activities but have never received treatment [21]. This study forms part of a larger study called SCAALA-Esmeraldas, which is examining the risk factors associated with differences in the prevalence of asthma and other allergic diseases in children from rural and migrant urban populations in Esmeraldas Province [42].

To evaluate the methods discussed in this paper, we analyzed data from a simple random sample of 2000 children from the original study. Here we are interested in investigating the effect of ivermectina on the prevalence of *Trichuris trichiura *after adjusting for children's age and gender.

Data analysis was done using STATA v.8 and R v.2.6.0 software [43].

### Simulation Studies

To compare different methods for estimating confidence intervals for PR in clustered data using logistic regression with random effects, simulation studies were conducted with varying degrees of dependency, through the intraclass correlation coefficient (ICC), and levels of clustering (given by number and size of clusters). For each configuration, 1,000 samples were generated. We present the coverage probability (CP) of the Wald 95% confidence interval for the corresponding estimation method for each combination of ICC, number and cluster sizes.

The coverage probabilities (CP) represent the percentage of simulated datasets in which the corresponding confidence intervals contain the true PR. For the simulation studies conducted here, CP should be 95% to indicate that the method used for defining the confidence intervals is accurate.

We generated correlated binary outcomes through a random effects logistic model using the algorithm presented by Moineddin, Matheson and Glazier [22]. The following steps were implemented to simulate data sets:

1. Set up values for fixed parameter *β *(the effect of covariates on the outcome), number and size of the clusters, and ICC.

2. Generate a dichotomous independent variable *X*_1*j *_representing an intervention for each data unit. The number of clusters was the same in each intervention group.

3. Generate a continuous independent variable *X*_2*ij *_from a Normal(0,1) distribution.

4. Generate a normal variable, such that for given cluster j, *u*_*oj *_~ *N*(0,$\sigma_{u}^{2}$), where *u*_*oj *_and *u*_*oj*' _are independent for *j *≠ *j*'. The intraclass correlation coefficient (ICC) [22] is defined by

$$ICC=\frac{\sigma_{u}^{2}}{\sigma_{u}^{2}+\pi^{2}/3}$$

5. Calculate *p*_*ij *_= *E*(*Y*_*ij *_|*X*_1*j*_, *X*_2*ij*_, *u*_*oj*_) using a random effects logistic model, such that

$$p_{ij}=\frac{\exp(X^{\prime }\beta +u_{oj})}{1+\exp(X^{\prime }\beta +u_{oj})}$$

6. The correlated binary outcome (*Y*_*ij*_) for the *i*^*th *^subject of the *j*^*th *^cluster is generated by a Bernoulli distribution with probability *p*_*ij*_

In the simulations, we considered 15, 30 and 100 clusters of sizes(*m*) 10 and 30. The ICC was defined to be 0.03, 0.29 and 0.71. The bootstrapping procedure took into account the clustering of the data. The simulation studies were implemented using R version 2.6.0 software [43].

## Results

### Data Analysis

#### SCAALA-Salvador Study

This analysis included data from 1087 children, aged 4 to 12 years-old, with 81.0% being 8 years-old or younger, 47.1% being girls and 26.7% with asthma. Among the mothers, 30.7% completed high school or college and 37.4% had probable mental health problems. For modeling, age was centered in its mean value. We estimated the effect of maternal mental health status on asthma occurrence using random effects logistic regression, considering two standardization methods and three approaches for getting the confidence intervals for PR. Results are presented in Table 1.

**Table 1:** Comparison of prevalence ratio (PR) estimates using random effects logistic regression: Impact of maternal mental health on child's asthma in Brazil.

Standardization Method	Random Effects Logistic Regression			
	
	PR	95% CI		
		
		Bootstrap-Normal	Bootstrap-percentiles	Delta
Conditional	1.52	(1.11;1.79)	(1.28;1.99)	(1.25;1.85)
Marginal	1.54	(1.18;1.80)	(1.32;1.93)	(1.24;1.91)

The adjusted odds ratio is 1.87 (95%CI = 1.41, 2.47), which is larger than the estimated prevalence ratio (PR = 1.52–1.54, depending on the standardization procedure). When estimating PR using conditional standardization, we specified a mean age of 6.8 years, the reference groups as boys and educational level of mothers less than elementary school. Based on the results of the conditional standardization, the prevalence of asthma, if the child is a boy aged 6.8 years, with mother with low educational level and mental health problem, is about 52% greater compared to the prevalence of the *same *boy aged 6.8 years having asthma, with mother with low educational level and no evidence of mental problem. On the other hand, if we choose a marginal standardization we can say that the prevalence of asthma assuming that all children in the study have mothers with mental health problems is 54% larger than the prevalence of asthma assuming that no children in the study have mothers with mental health problems. Note that the 95% bootstrap confidence intervals are wider than those obtained from the delta method when considering conditional standardization.

Robust Poisson regression and robust log-binomial regression were also implemented. The results obtained using the robust Poisson model (PR = 1.54,95%CI = 1.22,1.94) were very close to those obtained from the random effects logistic regression. Convergence was not achieved using the log-binomial model.

#### SCAALA-Ecuador Study

We analyzed data from 2000 children aged 6 to 16 years-old, of which 15.2% were aged 6–7 years, 23.5% 8–9 years, 24.3% 10–11 years, 21.7% 12–13 years, and 15.5% 14–16 years. Fifty-eight percent of the children were boys and 46.5% had received ivermectin. To evaluate the association between infection with *Trichuris trichiura *and ivermectin, we considered a random effects logistic model. We modelled the occurrence of infection as a function of ivermectin treatment, adjusting by gender and age. Age was centered in its mean value. The prevalence of infection was 57.9%. As expected in such scenarios, the odds ratio overestimated the effect of treatment (OR = 0.07 [95%CI = 0.05; 0.11]) compared to the prevalence ratio (PR = 0.33 [95%CI = 0.27; 0.42], using conditional standardization and delta method). The bootstrap confidence intervals based on normal theory were narrower than those obtained through delta method for the random effects logistic model in this application (Table 2).

**Table 2:** Estimation of prevalence ratio of Trichuris using standard and random effects logistic regression, and robust Poisson model:    Effectiveness of a health program in Ecuador.

Regression Models		PR	95% CI
Standard Logistic		0.38	
	Delta Method		(0.34;0.42)
	Bootstrap Method		(0.34;0.42)
Random Effects Logistic		0.33	
	Delta Method		(0.27;0.42)
	Bootstrap Method		(0.30;0.39)
Robust Poisson		0.38	(0.31;0.47)

The estimated PR for *T. trichiura infection *using robust Poisson was 0.38 [95%CI = 0.31; 0.47]. These results indicated a reduction of approximately 62% in the prevalence of *T. trichiura *infection in children treated with ivermectin of the same age and gender compared to untreated children. Convergence was not achieved for analysis of this data using log-binomial models.

For these data, the intraclass correlation coefficient (ICC) was 0.415, indicating an important effect of clustering that should be considered in the analysis. Standard logistic regression models tend to underestimate the standard errors for PRs compared to random effects logistic regression models. In general, the confidence intervals obtained in the random effects logistic regression are wider than those from the standard logistic regression (Table 2).

### Results of Simulation Studies

The findings of the simulation studies comparing the coverage probability (CP) of the Wald 95% confidence interval obtained through delta method and clustered bootstrap for random effects logistic model are shown in Table 3. The prevalence of disease for each of the configurations was between 55% and 60%, with a PR of 1.52.

**Table 3:** Coverage probability of the Wald 95% confidence interval of PR for delta method and bootstrap varying the degree of correlation, number and size of clusters.

Sample Size	ICC = 0.03		ICC = 0.29		ICC = 0.71	
	
	Delta	Bootstrap	Delta	Bootstrap	Delta	Bootstrap
Number of clusters = 10						
m = 10	94.7%	88.3%	92.7%	88.0%	95.0%	87.3%
m = 30	93.7%	93.0%	91.7%	88.3%	92.0%	89.0%
Number of clusters = 30						
m = 10	95.3%	94.0%	90.3%	91.0%	93.3%	92.0%
m = 30	92.3%	90.0%	94.0%	93.3%	94.0%	93.6%
Number of clusters = 100						
m = 10	94.3%	94.3%	92.7%	94.0%	91.8%	92.5%
m = 30	93.9%	92.9%	95.7%	95.3%	95.0%	94.3%

The results suggest that the delta method outperforms the bootstrap method, especially when the number of clusters is small. For instance, considering 10 clusters of size 10, the CP's of the Wald 95% confidence interval were 94.7% and 88.3%, respectively, for delta and bootstrap methods, when ICC equals to 0.03, and 95.0% and 87.3% when ICC equals to 0.71.

Standard logistic regression (no adjustment for clustering) performed poorly, particularly when increasing the number of clusters and increasing correlation between individuals within clusters. Considering 50 clusters of size 10 the CP dropped from 93.1–82.3%, when ICC equals 0.03, to 46.3–41.8%, when ICC equals to 0.71, for delta and bootstrap methods, respectively [data not shown].

The results for the comparison of logistic and Poisson random effects models are presented in Table 4. The delta method was used to obtain the 95% confidence intervals for PR for the random effects logistic model. The logistic model generally performed better than the Poisson model. Performance of the random effects Poisson model for estimating PR declined when there was a high degree of within-cluster correlation (ICC = 0.71) and with increasing number of clusters (k).

**Table 4:** Coverage probability of the Wald 95% confidence interval of PR using random effects logistic and Poisson model varying the degree of correlation and number of clusters of size 10.

ICC and Numb. clusters (k)	Random effects logistic model	Random effects Poisson model
ICC = 0.03		
k = 15	95%	98%
k = 30	94%	95%
k = 50	96%	89%
ICC = 0.29		
k = 15	92%	93%
k = 30	93%	87%
k = 50	94%	75%
ICC = 0.71		
k = 15	91%	85%
k = 30	93%	81%
k = 50	92%	73%

## Discussion

A major advantage of the odds ratio is that it can be estimated for all study types. However, investigators should avoid interpreting odds ratios as an approximation to prevalence ratios when the prevalence of the event of interest is high (greater than 10%). In such situations, the odds ratio generally overestimates the prevalence ratio. The importance of differences in the interpretation of the OR compared to PR/RR, particularly when prevalence is high, has been discussed by others [3,11,16].

If the adjusted prevalence ratio (PR) is the measure of interest, logistic regression is one of the approaches that can be used for its estimation [10,16]. However, the choice of standardization procedure may affect the point estimates and, most importantly, its interpretation. To our knowledge, there are few reports discussing implications of the choice of standardization for the interpretation of PR in the context of logistic regression [16]. The most recent effort to discuss this issue was done by Localio and colleagues (2007), in which the standardization procedure is linked to the question of interest. In contrast to OR, which is computed regardless of the values of other covariates, the calculation of PR using logistic regression is dependent on the fixed levels of covariates included in the model. Thus, a clear interpretation of PR depends on the definition of the reference values used on the computational procedure.

There is also no consensus about the the best way to interpret regression coefficients in the the context of random effects models. Some authors interpret the fixed regression coefficients similarly to the usual logistic regression model, conditioning on the random effects [27-29]. When modeling explicitly the source of heterogeneity in the logistic regression with random effects, the fixed regression parameters should be interpreted as effects of covariates on a typical subject in the study [30,44]. Thus, as an illustration using our application regarding impact of ivermectin in the prevalence of *Trichuris *infection, the estimated PR using logistic model with random effects represents the ratio of the probability of a given child having *Trichuris *infection if he/she receives ivermectin compared to the probability that the *same *child having *Trichuris *infection if he/she does not receive treatment. In this way the PR is adjusted for unobserved individual characteristics.

Alternatively, population-averaged estimates for the regression coefficients can be obtained using approximate formulae as suggested by Zeger and colleagues (1988), which can be interpreted in terms of the response averaged over the population [45]. In some situations, however, the subject specific interpretation is of more interest than its average effect on a population as a whole [46]. Another approach was proposed by Larsen and colleagues (2000), who discussed the interpretation of both fixed and random effects parameters in the context of logistic regression with random effects [27]. They proposed a measure for the fixed effect called median odds ratio (MOR) in order to take into account the fact that, in practice, the procedure of conditioning in the random effects is unrealistic because the random effects are unobservable.

The confidence intervals for prevalence ratio using logistic regression should be defined using appropriate approaches, such as delta and bootstrap methods. Other methods discussed in the literature, such as the substitution method [2], have been shown to have theoretical limitations leading to unsatisfactory statistical performance [10,16]. The use of delta and bootstrap methods have been discussed in the literature for situations where the observations are uncorrelated. In such cases, the performance of these methods seems to be equivalent.

Other model-based approaches that have been commonly used to estimate PR are the Poisson and log-binomial models [3,10,16-18]. The main advantage of these methods is the direct estimation of PR and its confidence intervals [47]. At the same time, both models can present estimation problems due to restrictions to avoid predicting probabilities out of interval [0,1]. When this happens, the model does not converge. There has been no consensus about the best model-based approach for estimating PR. Barros and Hirakata (2003) suggested that more than one modeling strategy should be used to evaluate the robustness of the results. A shortcoming of this strategy is that different models imply different relationships between the outcome and covariates, even when the same covariates are included in the model. Furthermore, identification of interaction effects may differ across models.

All previous discussions about the estimation of PRs has been done in the context of independent observations. In this paper we have extended this discussion to include clustered design studies, in which the dependence between observations is taken into account. We used random effects logistic models to deal with intracluster correlation. We evaluated the performance of methods for defining confidence intervals through simulation studies with several levels of correlation between observations in the same cluster. For the scenarios considered here the delta method outperformed the clustered bootstrap method when there are data for a small number of clusters. However, for situations where size and number of clusters are large, they show equivalent performance. We also noticed a poorer performance of the Poisson model with random effects, especially with increasing level of clustering and number of clusters, and there were problems with convergence when the number of clusters was small.

## Conclusion

We illustrated the estimation of prevalence ratios using data from two studies with health-related outcomes in children and we observed major differences between estimated PR and OR in these studies. Therefore, we highlight the importance of avoiding interpreting odds ratios as prevalence ratios in many situations, particularly when the outcome is not rare. Based on the results of the simulation studies, we recommend the use of the logistic model with random effects for analysis of clustered data when there are at least 30 clusters of size greater or equal to 10. The choice of estimation method for the calculation of confidence intervals for PRs – delta or clustered bootstrap methods – should be based on study design.

## Competing interests

The authors declare that they have no competing interests.

## Authors' contributions

Studies concept and design: MLB, LR, PJC. Analysis and interpretation: MBBC, ALM, SC, CAT, LDA. Development and implementation of statistical methodology: CAT, RLF, NFO, LDA. Simulation Studies: CAT, RLF, NFO, LDA. Drafting of the manuscript: CAT, RLF, NFO, LDA. All authors critically revised and approved the final manuscript.

## Pre-publication history

The pre-publication history for this paper can be accessed here:


